# Prospects for investigating brain oxygenation in acute stroke: Experience with a non‐contrast quantitative BOLD based approach

**DOI:** 10.1002/hbm.24564

**Published:** 2019-03-12

**Authors:** Alan J. Stone, George W. J. Harston, Davide Carone, Thomas W. Okell, James Kennedy, Nicholas P. Blockley

**Affiliations:** ^1^ Wellcome Centre for Integrative Neuroimaging, FMRIB, Nuffield Department of Clinical Neurosciences University of Oxford Oxford UK; ^2^ Acute Vascular Imaging Centre, Radcliffe Department of Medicine University of Oxford Oxford UK

**Keywords:** Ischaemia, oxygen metabolism, stroke

## Abstract

Metabolic markers of baseline brain oxygenation and tissue perfusion have an important role to play in the early identification of ischaemic tissue in acute stroke. Although well established MRI techniques exist for mapping brain perfusion, quantitative imaging of brain oxygenation is poorly served. Streamlined‐qBOLD (sqBOLD) is a recently developed technique for mapping oxygenation that is well suited to the challenge of investigating acute stroke. In this study a noninvasive serial imaging protocol was implemented, incorporating sqBOLD and arterial spin labelling to map blood oxygenation and perfusion, respectively. The utility of these parameters was investigated using imaging based definitions of tissue outcome (ischaemic core, infarct growth and contralateral tissue). Voxel wise analysis revealed significant differences between all tissue outcomes using pairwise comparisons for the transverse reversible relaxation rate (*R*_2_ ′), deoxygenated blood volume (DBV) and deoxyghaemoglobin concentration ([dHb]; *p* < 0.01 in all cases). At the patient level (*n* = 9), a significant difference was observed for [dHb] between ischaemic core and contralateral tissue. Furthermore, serial analysis at the patient level (*n* = 6) revealed significant changes in *R*_2_ ′ between the presentation and 1 week scans for both ischaemic core (*p* < 0.01) and infarct growth (*p* < 0.05). In conclusion, this study presents evidence supporting the potential of sqBOLD for imaging oxygenation in stroke.

## 1 INTRODUCTION

Ischaemic stroke is characterised by restricted blood supply to regions of tissue that may ultimately result in infarction. However, the brain can tolerate a limited reduction in perfusion if tissue oxygenation levels can be preserved. Therefore, techniques to map brain oxygenation in the acute phase of stroke may help to identify viable tissue that requires intervention to minimise the final infarct volume (Astrup, Siesjö, & Symon, 1981). While Positron Emission Tomography (PET) is the current benchmark for imaging oxygen metabolism in acute stroke (Ackerman et al., 1981; Baron, 1999), it is not widely available in acute clinical settings.

Magnetic resonance imaging (MRI) is already widely used in the assessment of patients with acute stroke and has the potential to be a viable alternative to PET in measuring oxygen metabolism. Such measurements are made possible by the inherent sensitivity of the transverse MR relaxation rate (*R*_2_ *) to deoxyhaemoglobin. *R*_2_ * (= *R*_2_ + *R*_2_ ′) is composed of the irreversible (*R*_2_ ) and reversible (*R*_2_ ′) transverse relaxation rates with respect to a spin echo. As changes in *R*_2_ (and hence *R*_2_*) are known to be affected by numerous factors aside from tissue oxygenation (An et al., 2012; An et al., 2014 ), *R*_2_′ is predicted to have better specificity to baseline brain oxygenation (Yablonskiy & Haacke, 1994 ). This sensitivity has previously been exploited to demonstrate that alterations in *R*_2_′ are indicative of the final outcome of ischaemic tissue (Bauer et al., 2014; Geisler et al., 2006; Seiler et al., 2012; Siemonsen et al., 2008; Zhang, Zhang, & Chen, 2011 ). However, *R*_2_′ is dependent on both deoxyhaemoglobin concentration ([dHb]) and the DBV (Yablonskiy, 1998 ), resulting in ambiguity regarding the physiological origin of a measured *R*_2_ ′ alteration. The ability to separate [dHb] from *R*_2_′ would provide a quantitative physiological metric directly related to tissue oxygenation, and to achieve this, knowledge of the underlying DBV is required.

Dynamic susceptibility contrast (DSC) MRI has been used to improve the physiological interpretation of *R*_2_′ by providing cerebral blood volume (CBV) and cerebral blood flow (CBF) information allowing [dHb] to be estimated (Christen, Schmiedeskamp, Straka, Bammer, & Zaharchuk, 2012). These methods have previously been employed to investigate acute stroke (Gersing et al., 2015; Seiler et al., 2017a; Seiler et al., 2017b). However, DSC requires an exogenous contrast agent, which may limit the frequency with which scanning can be repeated and be contraindicated in some patients (Gulani, Calamante, Shellock, Kanal, & Reeder, 2017). In addition, DSC provides an estimate of the blood volume across all vascular compartments, while qBOLD requires a measurement of the volume occupied specifically by deoxygenated blood.

There are noncontrast based alternatives that allow the estimation of DBV and CBF. The quantitative‐BOLD (qBOLD) method is able to measure DBV by modelling the transverse MR signal decay in the presence of a vascular network (An & Lin, 2000; He & Yablonskiy, 2007). Arterial spin labelling (ASL) can be used to quantify CBF by inverting the magnetisation of arterial blood to act as a diffusible tracer (Alsop et al., 2015). These endogenous methods are particularly suitable for application in acute stroke as they can be acquired noninvasively in a clinically relevant manner (An et al., 2015; Lee et al., 2003).

Recently we proposed a refinement of the qBOLD method targeted at minimising confounding effects during data acquisition, rather than by postprocessing, which we term streamlined‐qBOLD (sqBOLD; Stone & Blockley, 2017 ). By minimising the influence of nuisance signals (macroscopic field inhomogeneities, MFIs; cerebral spinal fluid, CSF; and *R*_2_‐weighting) during image acquisition the application of the qBOLD model is simplified, improving the robustness of the resultant oxygenation maps. While measurements in healthy young subjects look promising, so far measurements have not been performed in more challenging clinical research applications. The aim of this study is to investigate the potential of sqBOLD to monitor regional changes in brain oxygenation following ischaemic stroke, and its relation to tissue outcome over time in a cohort of patients undergoing serial non‐contrast multi‐modal MR imaging, including ASL perfusion imaging.

## 2 MATERIALS AND METHODS

### 2.1 Patients

Patients with ischaemic stroke were recruited into a prospective observational cohort study regardless of age or stroke severity under research protocols agreed by the UK National Research Ethics Service committees (ref: 13/SC/0362). The following inclusion criteria were used to select patients for this analysis: DWI (*b* = 1,000 s/mm ^2^) lesion on the presenting scan and a follow up scan at either 24 hr or, preferably, 1 week following the initial scan to enable the definition of the regions of interest. Patients with lacunar stroke, as defined on DWI scan (lesions <15 mm in diameter), were excluded from this analysis. Nine patients met the criteria to be included in this analysis (Table 1). The median symptom onset to MRI time was 13 hr 49 min (range 2 hr 20 min – 1 day 4 hr 19 min, Table 1). Where possible, follow‐up scanning was performed at 2 hr, 24 hr, 1 week, and 1 month relative to the presentation scan.

**Table 1:** Patient characteristics

Patient	Stroke syndrome	Hemisphere	Sex	Age	NIHSS	Thrombolysed	Onset to scan	Follow‐up scans	Voxels in ROI (core, growth)	Mean motion score
P01	PACS	Right	F	69	6	Y	00:10:52	1 week, 1 month	59, 38	0.17
P02	POCS	Left	M	68	3	Y	00:04:11	2 hr, 1 week, 1 month	8, 49	0.18
P03	TACS	Left	M	79	14	Y	00:02:20	24 hr	1264, 2270	0.56
P04	POCS	Left	M	68	12	Y	00:19:33	24 hr	94, 225	0.62
P05	PACS	Right	M	78	4	N	01:04:19	24 hr (38 hr), 1 month	560, 167	2.12
P06	PACS	Right	M	87	19	Y	00:13:49	24 hr, 1 week	190, 97	0.20
P07	TACS	Left	M	77	22	Y	00:13:05	24 hr, 1 week	675, 5,725	0.24
P08	TACS	Right	F	74	10	Y (+ thrombectomy)	00:22:17	24 hr, 1 week	61, 688	0.44
P09	TACS	Right	F	80	17	Y	00:17:50	24 hr, 1 week	3237, 1619	0.13

NA = not available; NIHSS = National Institute of Health Stroke Scale; PACS = partial anterior circulation stroke; POCS = posterior circulation stroke; TACS = total anterior circulation stroke.

### 2.2 Image acquisition

Scanning was performed on a Siemens 3T Verio scanner (Siemens Healthineers, Erlangen, Germany) for all time points. The scanning protocol included the MRI techniques described in the following paragraphs.

Diffusion weighted imaging (DWI) with apparent diffusion coefficient (ADC) calculation was used to identify areas of cerebral ischaemia based on restricted diffusion caused by cell swelling (Le Bihan et al., 2001) (three directions, 1.8 × 1.8 × 2.0 mm, field of view = 240 mm^2^ , four averages, *b* = 0 and 1,000 s/mm ^2^, TR/TE = 9,000/98 ms, 50 slices, 2 min 53 s).

A T_1_‐weighted (T1w) MP‐RAGE was used for structural imaging and image registration (1.8 × 1.8 × 1.0 mm, field of view = 228 mm, TR/TE = 2040/4.55 ms, TI = 900 ms, 192 slices, scan duration 3 min 58 s).

A *T*_2_‐weighted FLAIR turbo spin echo was used to identify infarction on follow‐up scans (Harston et al., 2017) (1.9 × 1.9 × 2.0 mm, field of view = 240 × 217.5 mm^2^, TR/TE = 9,000/96 ms, TI = 2,500 ms, 58 slices, scan duration 2 min 8 s).

Vessel encoded Pseudo‐Continuous Arterial Spin Labelling (PCASL) was used for perfusion imaging and CBF calculation (Okell, Chappell, Kelly, & Jezzard, 2013; EPI readout, 3.4 × 3.4 × 4.5 mm, field of view = 220 × 220 mm, 24 slices, TR/TE = 4,080/14 ms, labelling duration = 1.4 s, postlabelling delays = 0.25, 0.5, 0.75, 1, 1.25, and 1.5 s, scan duration 5 min 55 s). Performing ASL with multiple postlabelling delays improves absolute quantification of CBF in the presence of delayed blood arrival times (Harston et al., 2017; Okell et al., 2013; Wang et al., 2013). This is in contrast to DSC perfusion weighted imaging (PWI) where prolonged time to peak (TTP) or mean transit time (MTT) are commonly used to define hypoperfused tissue, rather than CBF.

A FLAIR‐GASE acquisition was used to measure baseline brain oxygenation using the sqBOLD approach (Stone & Blockley, 2017) (96 × 96 matrix, field of view = 220 mm^2^, nine 5 mm slabs consisting of four 1.25 mm sub‐slices, 100% partition oversampling, 1 mm slice gap, TR/TE = 3,000/82 ms, TI_FLAIR_ = 1,210 ms, ASE‐sampling scheme τ_start_/τ_finish_/Δτ = −16/64/8 ms, scan duration 4 min 30 s). FLAIR‐GASE consists of three separate components, nulling of CSF partial volumes using FLuid Attenuated Inversion Recovery (FLAIR; Hajnal et al., 1992 ), direct measurement of *R*_2_′ using an asymmetric spin echo (ASE; Wismer et al., 1988) and minimisation of MFI using gradient echo slice excitation profile imaging (GESEPI; Yang, Williams, Demeure, Mosher, & Smith, 1998). The GESEPI technique compensates for residual magnetic field gradients (MFGs) in the slice direction that are not corrected by the manufactuer's standard 3D volume shim (Gruetter & Boesch, 1992). This is achieved by oversampling in the slice direction using a 3D acquisition. It has recently been shown that by combining GESEPI with the ASE technique (GASE) the effect of MFGs can be minimised in the majority of the brain (Blockley & Stone, 2016). However, GASE can only compensate MFGs below a critical threshold. Hence positioning of the imaging volume was focussed on the ischaemic region and slices were angeled away from areas of severe MFI. The combined FLAIR‐GESEPI‐ASE (FLAIR‐GASE) acquisition reduces confounding effects and when combined with quantitative modelling offers a streamlined qBOLD approach.

### 2.3 Post‐processing

All image analysis was performed using the Oxford Centre for Functional MRI of the Brain (FMRIB) Software Library (FSL) (Jenkinson, Beckmann, Behrens, Woolrich, & Smith, 2012) and MATLAB (Mathworks, Natick, MA). Post‐processing details of VEPCASL data to produce CBF maps have previously been described (Harston, Okell, et al., 2017; Okell et al., 2013).

For sqBOLD, preprocessing and parameter map calculation from the FLAIR‐GASE data is based on previously described methods (Stone & Blockley, 2017). In brief, the four 1.25 mm slices of each slab were averaged to produce a single 5 mm slice. The τ‐series was motion corrected using the FSL linear motion correction tool (MCFLIRT; Jenkinson, Bannister, Brady, & Smith, 2002) to the spin‐echo image. As an indicator of the severity of head‐motion that occurred during FLAIR‐GASE acquisition, the mean relative root mean square value was output from MCFLIRT, and henceforth described as the mean motion score. The spin‐echo image was brain extracted using the FSL brain extraction tool (BET; Smith, 2002) to create a binary mask of brain tissue and all remaining τ‐weighted volumes were brain extracted using this mask. The data were spatially smoothed using a Gaussian kernel with a full‐width half‐maximum that matched the in‐plane resolution (2.3 mm). This smoothing was chosen to reduce the impact of noisy voxels on the model fit without unduly reducing the spatial resolution of the resulting parameter maps.

Oxygenation parameters *R*_2_′, DBV and [dHb] were directly inferred from the sqBOLD data using the qBOLD model (Yablonskiy, 1998 ). To describe this model in simple terms, the mono‐exponential part of the signal decay (*τ* > 15 ms) is used to measure *R*_2_ ′ and the mismatch between a measured spin echo (*τ* = 0 ms) and the linear intercept of the mono‐exponential regime provides an estimate of the DBV. The ratio of *R*_2_′ and DBV is proportional to [dHb] (Equation (2) ). To estimate sqBOLD oxygenation parameters for each voxel, *R*_2_ ′ and DBV were organised into a vector of unknowns (*x*) in a linear system ( *A* ∙ *x* = *B*; Equation (1) ). *S*(*τ*) is the signal intensity of a given voxel for a spin‐echo displacement time (*τ*) and *τ*_1,2, … *n*_ refers to the range of spin‐echo displacement times acquired in the long *τ* regime (*τ* > 15 ms; Yablonskiy & Haacke, 1994 ). In this study *τ* values of 16, 24, 32, 40, 48, 56, and 64 ms were used. The first row of matrix *A* describes where *τ* = 0, which is insensitive to *DBV*.

(1) $$\left[\begin{array}{ccc} 0 & 0 & 1\\ 1 & -\tau_{1} & 1\\ 1 & -\tau_{2} & 1\\ \vdots & \vdots & \vdots \\ 1 & -\tau_{n} & 1 \end{array}\right]\left[\begin{array}{c} \mathrm{DBV}\\ {R_{2}}^{\prime }\\ \log\left(S_{0}\right)-\mathrm{TE}∙R_{2} \end{array}\right]=\left[\begin{array}{c} \log\left(S\left(0\right)\right)\\ \log\left(S\left(\tau_{1}\right)\right)\\ \log\left(S\left(\tau_{2}\right)\right)\\ \vdots \\ \log\left(S\left(\tau_{n}\right)\right) \end{array}\right]$$

The weighted least squares solution was then used to produce voxel‐wise estimates and standard deviation of *R*_2_ ′ and DBV. The model fit was inversely weighted for *τ*, with data being acquired at higher values of *τ* receiving less weighting in the fit, due to the increasing contribution of noise with increasing signal decay. This helps to account for the lower SNR in data acquired at longer *τ*‐values (Stone & Blockley, 2017). Parameter maps of [dHb] were calculated using Equation (2) , where DBV and *R*_2_′ were estimated as above and other parameters are known or assumed constants (Δχ_0_ = 0.264 × 10^−6^ (He & Yablonskiy, 2007 ), *κ* = 0.03 (McPhee & Hammer, 2009)).

(2) $$\left[\mathrm{dHb}\right]=\frac{3.{R_{2}}^{\prime }}{\mathrm{DBV}∙4∙\gamma ∙\pi ∙{\Delta \chi }_{0}∙\kappa ∙B_{0}}$$

Estimating [dHb] negates the requirement for an assumed or measured haematocrit, which is required in order to estimate the oxygen extraction fraction (OEF). The standard deviation of [dHb] was calculated by propagating the standard deviations calculated for *R*_2_′ and DBV.

### 2.4 Regions of interest

Binary masks of the presenting lesion were automatically generated by thresholding maps of ADC at 620 × 10^−6^ mm^2^/s (Purushotham et al., 2015). Initial clustering was performed using the FSL Cluster tool (http://fsl.fmrib.ox.ac.uk/fsl/fslwiki/Cluster). The ROI cluster was identified and smoothed (Gaussian kernel of standard deviation 1 mm) and followed by repeat cluster analysis. The small amount of smoothing applied to the initial binary cluster mask was performed to remove isolated noisy voxels during repeat clustering, resulting in better specificity of the ADC ROI to the presenting lesion. These automated ADC masks were inspected by a clinician to ensure their accuracy and manually corrected when necessary (Harston et al., 2015 ). An independent observer manually defined the final infarct ROI. This was preferentially performed using the 1‐week *T*_2_ ‐FLAIR image or, if unavailable, the 24 hr *b* = 1,000 s/mm ^2^ DWI image (Harston, Minks, et al., 2017).

The following tissue outcomes were used in the analysis and were defined from the infarct ROIs in the native space of the sqBOLD and ASL parameter maps.

- The ischaemic core is tissue common to both the presenting ADC lesion and final infarct.
- Infarct growth is tissue present in the final infarct that is not present in the presenting ADC lesion.
- The contralateral tissue is defined by a composite mask of the presenting and final infarct tissue mirrored to the contralateral side of the brain.

### 2.5 Registration

Registration of imaging modalities within a single time point was achieved using rigid body registration (6 degrees of freedom [DOF]) (Jenkinson & Smith, 2001 ). Between time point registration was performed using nonlinear registration of the *T*_1_‐weighted structural scans to limit potential error introduced by edema (Harston, Minks, et al., 2017 ). To create contralateral ROIs, the infarct masks were mirrored in standard space following nonlinear registration of the *T*_1_‐weighted image to a standard atlas (MNI152; Mazziotta et al., 2001 ). At each time point, the FLAIR‐GASE spin‐echo image (*τ* = 0 ms) was registered (6 DOF) to the *T*_1_ ‐structural using the *b* = 0 s/mm ^2^ DWI image as an intermediate registration step. For the ASL data, an unsubtracted reference volume was registered (6 DOF) directly to the *T*_1_‐structural image.

### 2.6 Regional analysis

For the MRI data acquired during presentation and follow‐up scanning, voxel values of *R*_2_′, DBV, [dHb], and CBF were extracted from the native space of the acquired parameter maps using the ROI definitions of ischaemic core, infarct growth and contralateral tissue.

A voxel‐level analysis of the presenting imaging data was conducted by pooling voxel values of *R*_2_ ′, DBV, and [dHb] across all patients for each tissue outcome ROI. This analysis enables the sensitivity of the sqBOLD parameters to different tissue outcomes. For each parameter (*R*_2_′, DBV, and [dHb]), differences between the voxel‐value‐distributions from the tissue outcome ROIs on presentation (ischaemic core, infarct growth, and contralateral tissue) were tested. To test the null hypothesis of no difference between the voxel‐value‐distributions from the tissue outcome ROIs a Kruskal–Wallis test was used. The Kruskal–Wallis test is a version of the classical one‐way ANOVA that does not require the assumption of normally distributed data. This non‐parametric test was chosen as the distribution of [dHb] values in healthy grey matter have previously been found to be nonnormally distributed (Stone & Blockley, 2017). On rejecting the null hypothesis, post hoc pairwise comparisons between the different tissue outcome ROIs were performed using the Tukey–Kramer method (honest significant difference test) to consider which pairs were significantly different. The Tukey–Kramer method controls for such multiple comparisons.

A patient‐level analysis of the presenting imaging data was conducted by calculating the median *R*_2_′, DBV, [dHb], and CBF for each of the tissue outcome ROIs in each patient for presenting scans. For the median values extracted across the patient group, the null hypothesis of no difference between the three tissue outcomes was tested using a two‐way ANOVA. Again if the null hypothesis was rejected for a given parameter, post hoc pairwise comparisons were performed using the Tukey–Kramer method.

To aid the development of suitably powered follow on studies, the results of these experiments were used to calculate the effect size for each measure. Cohen's *d* was chosen to measure the differences between means when comparing each of the pairs of tissue outcome ROIs. Since these data are paired, *d* was calculated from the mean difference and standard deviation of differences.

To investigate the evolution of sqBOLD parameters in follow‐up scans, the median *R*_2_ ′, DBV, [dHb], and CBF were calculated for each of the tissue outcome ROIs in each patient for the presenting and 1 week scans. Median parameter values in the ischaemic core and infarct growth ROIs were normalised within scan to the contralateral tissue ROI. Two tailed paired *t*‐tests were performed to test the hypothesis that the mean difference between time points is zero.

## 3 RESULTS

### 3.1 Group characteristics

Nine consecutive large volume stroke patients met the criteria for inclusion in this analysis. The median National Institute of Health Stroke Scale at presentation was 12 (range 3–22). Eight patients received intravenous (IV) thrombolysis with one of those patients also undergoing thrombectomy (Table 1). Infarct growth was observed in all nine patients. Patient demographics, including details of the number of voxels included in the ischaemic core and infarct growth ROIs in the space of the FLAIR‐GASE image space, are provided in Table 1.

### 3.2 Comparison of presentation scans and tissue outcome

**Figure 1:**
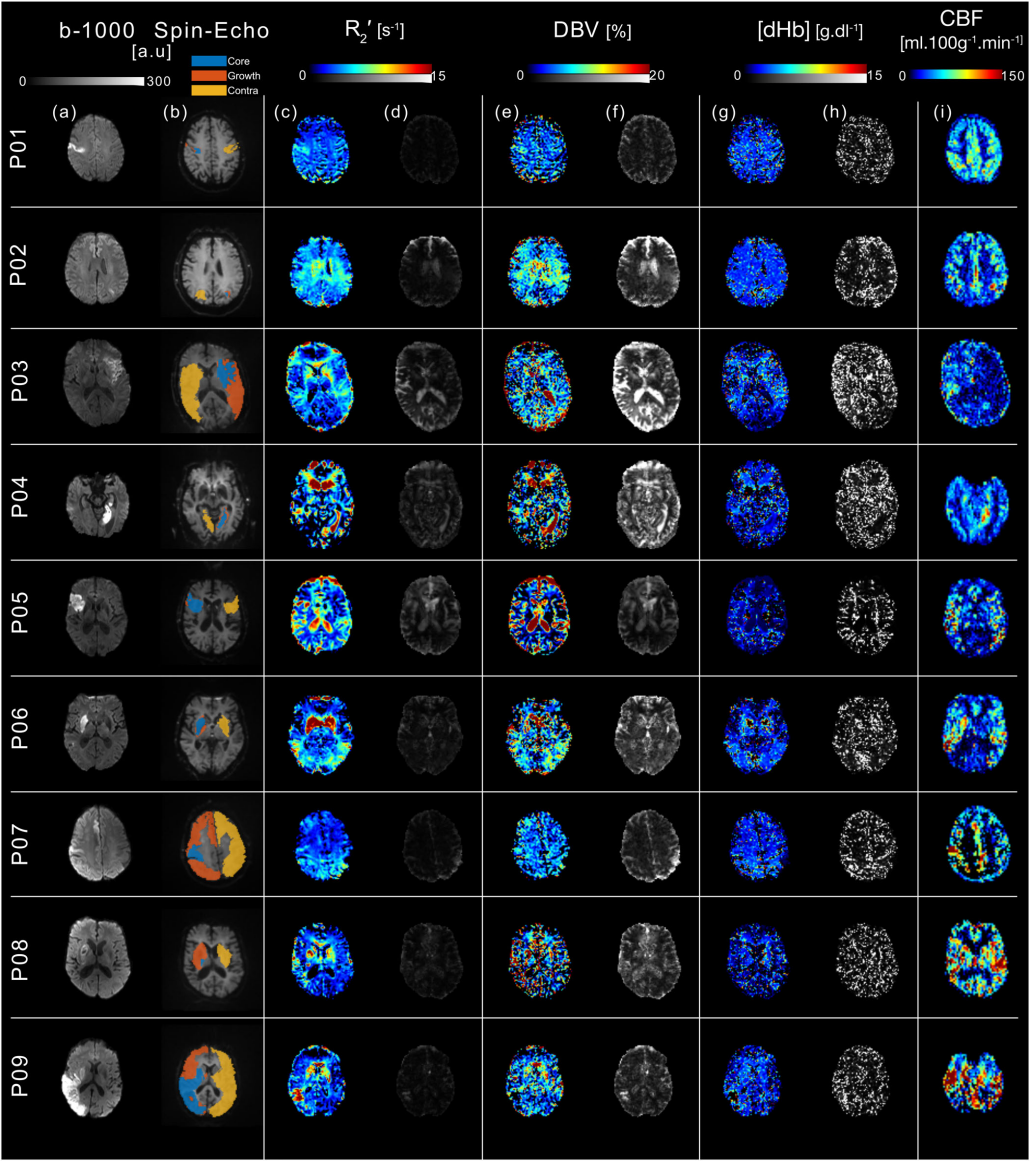
Data acquired during initial MRI scanning for all patients included in the study (*n* = 9). (a) DWI (*b* = 1,000 s/mm^2^), (b) asymmetric spin‐echo (*τ* = 0 ms), (c–h) sqBOLD oxygenation and (i) ASL blood flow maps are presented for a single axial slice in each stroke patient. All images are presented in their native acquisition space. Core (blue), growth (orange), and contralateral (yellow) tissue outcome ROIs are displayed on the spin‐echo image of the sqBOLD acquisition (b). Streamlined‐qBOLD parameter maps (*R*_2_′(c), DBV(e), and [dHb](g)) are presented alongside standard deviations on the parameter estimates calculated from the least‐squares fitting for (d) *R*_2_′, (f) DBV, and (h) [dHb] [Color figure can be viewed at http://wileyonlinelibrary.com]

To demonstrate the potential of sqBOLD for quantitatively mapping oxygenation in a clinical research setting, data acquired during the initial MRI scan session are displayed in Figure 1 for all patients (*n* = 9). For each patient, oxygenation (sqBOLD) and blood flow (ASL) parameter maps are presented alongside diffusion (DWI, *b* = 1,000 s/mm ^2^ ) images. Core (blue), growth (orange), and contralateral (yellow) tissue outcome ROIs are displayed on the sqBOLD spin‐echo image. As a measure of the error in the oxygenation parameters, maps of the standard deviation of *R*_2_′, DBV and [dHb] are provided. Higher error is noticeable in patients with more severe head motion during acquisition. For example, patient P05 displays the highest mean motion score and shows the largest standard deviation in the oxygenation parameters compared with patient P09, who moved the least. The mean motion scores estimated during motion correction for the presentation scans are listed in Table 1. It can also be seen that high DBV in the ventricles coincides with high DBV standard deviation and a large mean motion score. In contrast, regions of tissue generally show low standard deviation across oxygenation parameters, which does not appear to vary across tissue outcome ROIs. This demonstrates a better fit to the qBOLD model (Equation (1)) in these regions.

**Figure 2:**
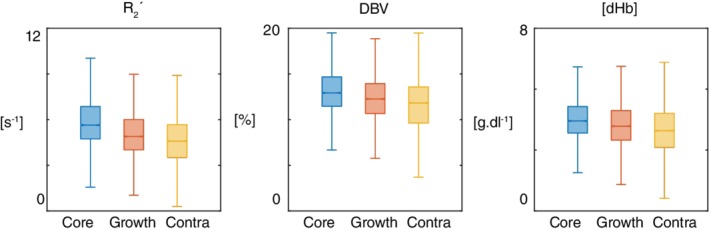
Box and whisker plot showing voxel‐level analysis of the tissue outcome from presenting time points. For each parameter (*R*_2_′, DBV, and [dHb]) at presentation, the null hypothesis was rejected suggesting that the voxel‐value‐distributions differed between the tissue outcome ROIs (Kruskal–Wallis test, *p* < 0.001 in all cases). Post hoc multiple comparisons analysis showed statistically significant pairwise differences between the tissue outcome ROIs (ischaemic core and contralateral tissue; infarct growth and contralateral tissue; ischaemic core and infarct growth; *p* < 0.01 in all cases) for each parameter [Color figure can be viewed at http://wileyonlinelibrary.com]

Figure 2 presents the voxel‐level analysis as the median and interquartile ranges of each tissue type at the presenting time point via box and whisker plots (whisker length 1.5 × interquartile range, outliers outside the whisker length are not shown).

Each parameter (*R*_2_ ′, DBV, and [dHb]), as measured at presentation, differed significantly between the tissue outcome ROIs (Kruskal–Wallis test, *p* < 0.001). Post hoc pairwise comparisons of each parameter in the different ROIs (ischaemic core and contralateral tissue; infarct growth and contralateral tissue; ischaemic core and infarct growth) revealed differences between all ROIs for each parameter (*p* < 0.01 in all cases). A more detailed voxel‐level investigation of CBF in a similar patient cohort has been published elsewhere (Harston, Okell, et al., 2017).

**Figure 3:**
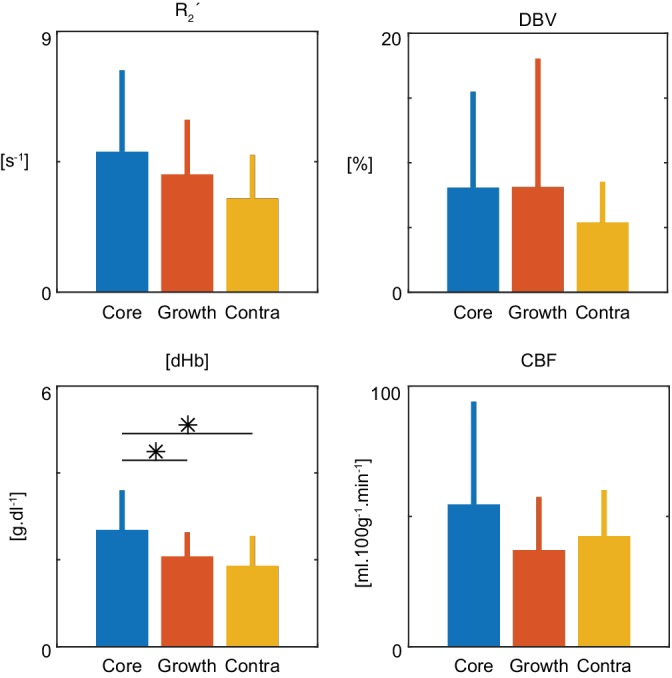
Patient‐level, group average (± standard deviation) parameters showing tissue outcome from presenting time points. Using a two‐way ANOVA the null hypothesis was rejected in the case of [dHb] (*p* = 0.0001). Post hoc, multiple comparisons testing showed a significant difference between [dHb] measured in the core compared to growth (*p* = 0.0016) and core compared to contralateral tissue (*p* = 0.0001). No significant difference was found between [dHb] measured in growth and contralateral tissue (*p* = 0.3207) [Color figure can be viewed at http://wileyonlinelibrary.com]

Figure 3 presents the patient‐level analysis of the presenting imaging data. Group‐average (mean ± standard deviation) baseline brain oxygenation and perfusion measurements are shown for each of the tissue outcome ROIs. A significant difference between tissue outcomes was detected for [dHb] (two‐way ANOVA, *p* < 0.0001), but not for the other parameter maps (*R*_2_ ′, DBV, and CBF). Post hoc pairwise comparisons of the tissue outcomes for [dHb] revealed a significant difference (*p* < 0.01) between ischaemic core and contralateral tissue and between ischaemic core and infarct growth. However, the patient‐level measures demonstrate a heterogeneity of responses across the group (Table 2). For example, both hypo‐ and hyperperfusion was observed for measurements of CBF. The effect size was greatest for [dHb] when making pairwise comparisons between each of the tissue outcomes and the contralateral ROI (Table 3).

**Table 2:** Parameter map estimates for each patient and tissue outcome ROI at presentation

Patient	*R*_2_′ (s^−1^)			DBV (%)			[dHb] (g.DL^−1^)			CBF (mL·100g^−^1·min^−1^)		
	Core	Growth	Contra	Core	Growth	Contra	Core	Growth	Contra	Core	Growth	Contra
P01	5.3	5.1	3.2	7.2	6.5	3.7	2.7	2.6	2.2	9.3	12.8	58.8
P02	3.6	3.2	3.5	4.2	4.1	5.1	3.6	2.8	2.3	23.4	19.3	29.7
P03	5.1	4.7	3.2	6.9	7.0	3.1	2.7	2.3	2.0	11.1	14.8	37.5
P04	3.5	4.3	2.4	6.5	3.6	4.5	1.8	1.9	1.6	67.6	54.3	40.5
P05	8.4	9.6	4.1	2.7	3.4	1.3	1.1	1.0	0.5	16.0	22.0	17.3
P06	12.4	4.3	7.5	8.8	6.0	6.6	4.1	2.6	2.7	85.1	35.1	25.9
P07	3.3	2.4	2.3	4.2	3.9	2.9	2.6	1.9	1.6	110.8	53.2	44.7
P08	2.9	4.1	4.3	2.5	4.2	5.3	3.3	2.0	2.5	90.3	60.1	53.4
P09	3.9	2.8	2.1	5.3	3.6	4.1	2.4	1.7	1.3	77.5	61.8	74.2
Mean	5.4	4.5	3.6	5.4	4.7	4.1	2.7	2.1	1.9	54.6	37.1	42.5
*SD*	3.1	2.1	1.7	2.1	1.4	1.6	1.0	0.6	0.7	39.5	20.4	17.7

**Table 3:** Effect size between tissue outcome ROIs calculated using Cohen's d to consider differences between means

Parameter	Core vs. contra	Core vs. growth	Growth vs. contra
*R*_2_′	0.92	0.31	0.39
DBV	0.57	0.02	0.38
[dHb]	2.12	1.14	0.64
CBF	0.31	0.74	0.28

### 3.3 Comparison of presentation and follow up scans

**Figure 4:**
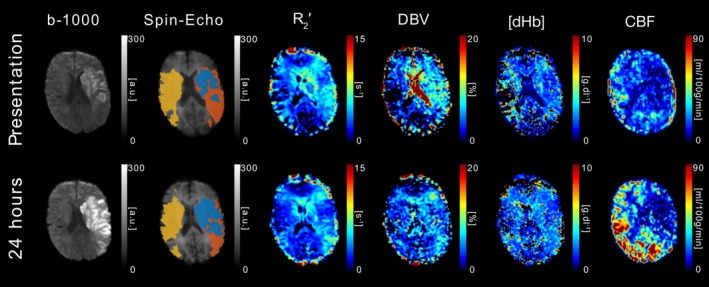
DWI (*b* = 1,000 s/mm^2^), sqBOLD parameter maps (*R*_2_′, DBV, and [dHb]) and CBF maps are presented for a single axial slice in an example patient. Core (blue), growth (orange), and contralateral (yellow) tissue outcome ROIs are displayed on the spin‐echo image of the sqBOLD acquisition. Patient P03 (male, 79 years old, NIHSS = 14, IV thrombolysis at 1 hr 24 min postonset) was scanned on presentation (2 hr 20 min postonset) and again at 24 hr [Color figure can be viewed at http://wileyonlinelibrary.com]

**Figure 5:**
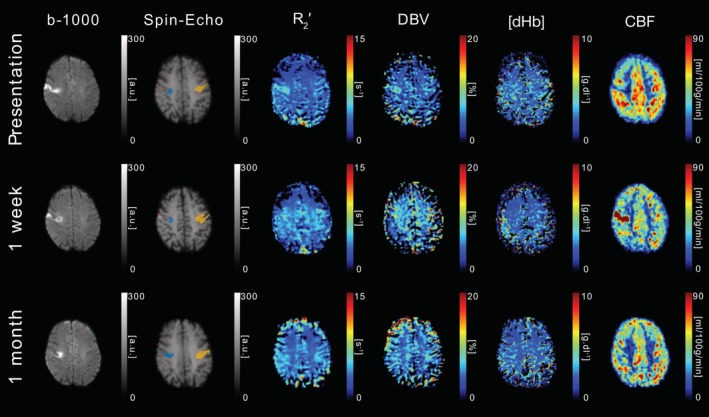
DWI (*b* = 1,000 s/mm^2^), sqBOLD parameter maps (*R*_2_′, DBV, and [dHb]) and CBF maps are presented for a single axial slice in an example patient. Core (blue), growth (orange), and contralateral (yellow) tissue outcome ROIs are displayed on the spin‐echo image of the sqBOLD acquisition. Patient P01 (female, 69 years old, NIHSS = 6, IV thrombolysis at 2 hr 31 min postonset) was scanned on presentation (13 hr 49 min postonset) and again at 24 hr and 1 week postinitial scan [Color figure can be viewed at http://wileyonlinelibrary.com]

**Figure 6:**
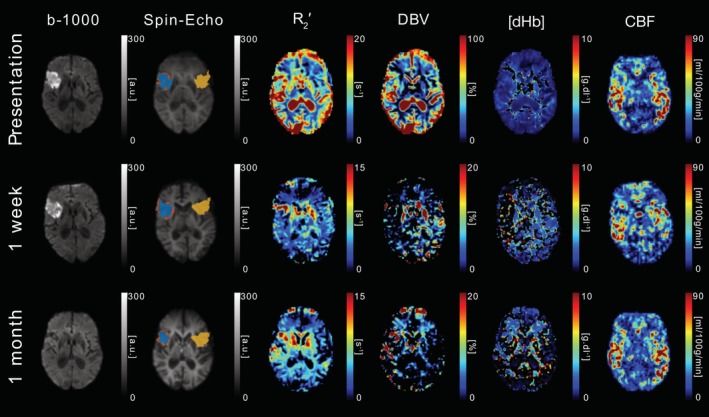
DWI (*b* = 1,000 s/mm^2^), sqBOLD parameter maps (*R*_2_′, DBV, and [dHb]) and CBF maps are presented for a single axial slice in an example patient. Core (blue), growth (orange), and contralateral (yellow) tissue outcome ROIs are displayed on the spin‐echo image of the sqBOLD acquisition. Patient P05 (male, 78 years old, NIHSS = 4, no IV thrombolysis) was scanned on presentation (28 hr 20 min postonset) and again at 38 hr and 1 month postinitial scan [Color figure can be viewed at http://wileyonlinelibrary.com]

**Figure 7:**
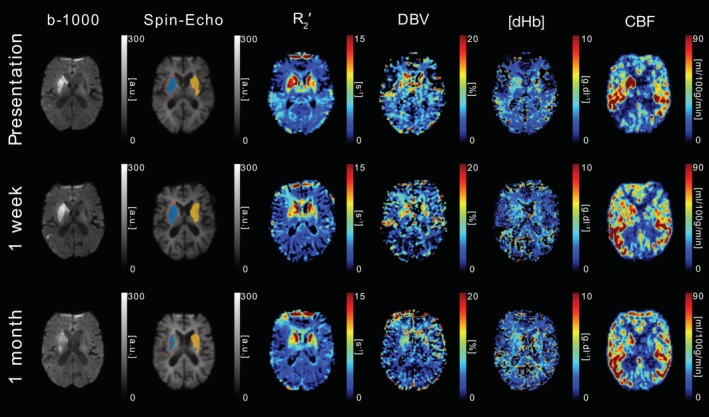
DWI (*b* = 1,000 s/mm^2^), sqBOLD parameter maps (*R*_2_′, DBV, and [dHb]) and CBF maps are presented for a single axial slice in an example patient. Core (blue), growth (orange) and contralateral (yellow) tissue outcome ROIs are displayed on the spin‐echo image of the sqBOLD acquisition. Patient P06 (male, 87 years old, NIHSS = 19, IV thrombolysis at 3 hr 28 min postonset) was scanned on presentation (13 hr 49 min postonset) and again at 24 hr and 1 week postinitial scan [Color figure can be viewed at http://wileyonlinelibrary.com]

The pertinent features of the sqBOLD technique as applied to acute stroke are illustrated through four example patients (Figures 4, 5, 6, 7). Firstly, the use of sqBOLD to investigate brain oxygenation over multiple time points is demonstrated in two patients and, secondly, two important confounding effects are considered through further examples. Lastly, the potential of serial imaging is investigated at the patient level by comparing the presenting data with images acquired at 1 week.

The images in Figure 4 were acquired from patient P03 on presentation (2 hr 20 min postonset) and 24 hr after presentation. This patient received IV thrombolysis at 1 hr 24 min post‐onset. On presentation, a lesion is clearly evident on the *b* = 1,000 s/mm ^2^ map. The presenting *R*_2_ ′ parameter map shows a large region of elevated *R*_2_ ′ in the area surrounding the presenting DWI lesion, which corresponds to a region of reduced CBF in the presenting CBF map. At the follow up imaging time point (24 hr), the DWI lesion has grown to include the region that was elevated on the presenting *R*_2_ ′ parameter maps, while the region of reduced CBF remains similar. Elevated *R*_2_ ′ is indicative of an increase in the presence of deoxyhaemoglobin in this region, which is driven by increases in DBV and/or [dHb] as seen in the accompanying parameter maps. In contrast to the large elevated region of *R*_2_ ′ there is also a region of reduced *R*_2_ ′ that approximately coincides with the presenting DWI lesion, which is indicative of a reduced amount of deoxyhaemoglobin. Nevertheless, the median *R*_2_′ extracted from the ischaemic core ROI, which is defined based on the presenting DWI, is still greater than the infarct growth ROI (Table 2 ). The measure of *R*_2_′ in this region appears to decrease between the presenting and follow‐up imaging time points.

Figure 5 displays data from patient P01 on presentation (10 hr 52 min postonset) and at 1 week and 1 month after presentation. This patient received IV thrombolysis at 2 hr 31 min post‐onset. At presentation, a well defined lesion is visible in the *b* = 1,000 s/mm ^2^ images. This is paralleled by similarly well defined regions of elevated *R*_2_ ′ and reduced CBF. An elevation in DBV can also be seen. At 1 week *R*_2_′ appears to have normalised, but now hyperperfusion is seen where previously hypoperfusion had been present. Finally, CBF is observed to be normalised by the 1 month scan.

Figure 6 shows images acquired from patient P05 who did not receive IV thrombolysis and was scanned on presentation (28 hr 20 min postonset), with follow up scanning performed at 38 hr and 1 month after presentation. Elevated *R*_2_′ is observed in regions expected to contain CSF at presentation. This is consistent with a failure of the CSF nulling FLAIR preparation. This is most likely due to patient motion since this patient recorded the highest mean motion score of 2.12, which compares with the 1 month scan where CSF signal appears nulled and a mean motion score of 0.86 was recorded. Maps of CBF demonstrate a heterogenous pattern of perfusion in the ischaemic region and appear to be relatively consistent between imaging time‐points.

Figure 7 shows images acquired from patient P06 scanned on presentation (13 hr 49 min postonset), with follow up scanning performed at 24 hr and 1 week after presentation. This patient received IV thrombolysis at 3 hr 28 min post‐onset. On presentation, there is an obvious deep grey matter lesion on the affected side of the brain that is clearly visible on the *b* = 1,000 s/mm ^2^ image, which corresponds with a region of elevated CBF compared with the mirrored contralateral ROI. However, the presenting *R*_2_ ′ parameter maps show bilateral elevations in *R*_2_′ on both the affected and unaffected sides, although to a larger degree in the ischaemic core (Table 2 ). These deep grey matter structures are known to have high iron content and the presence of this iron causes an elevation in *R*_2_ ′ that is unrelated to oxygenation and confounds the oxygenation measurement that is made within this region. Similarly, elevated *R*_2_′ regions are also observed in the growth ROI of patient P08, which is partially within this deep grey matter region (Figure 1). This highlights the importance of considering sources of susceptibility other than deoxyhaemoglobin in the locality of the region of interest. Despite this [dHb] appears to be elevated in the affected hemisphere compared with the unaffected hemisphere (Table 2).

**Figure 8:**
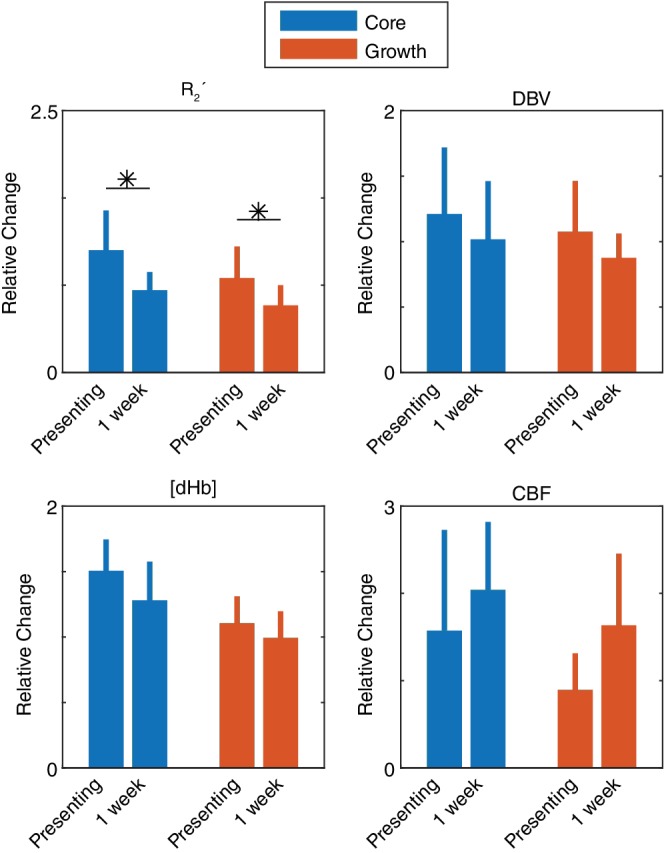
Comparison of measurements made during presenting and 1 week stroke‐onset‐to‐scan‐times (*n* = 6). For each parameter (*R*_2_′, DBV, [dHb], and CBF), measurements made in core and growth ROIs are normalised to contralateral tissue and group averaged (± standard deviation). Significant decreases in *R*_2_′ were detected in both core (*p* = 0.049) and growth (*p* = 0.006) after 1 week (paired *t*‐test). *p < 0.05 [Color figure can be viewed at http://wileyonlinelibrary.com]

Figure 8 compares measurements made at presentation with those made at the follow up scan at 1 week, which was available in 6 of the 9 patients. The null hypothesis was rejected for the *R*_2_ ′ measurements in the ischaemic core (paired *t*‐test, *p* < 0.05) and infarct growth (paired *t*‐test, *p* < 0.01) ROIs. In both cases *R*_2_′ is reduced at 1 week compared with presentation, which is consistent with increased blood oxygenation. This increase in blood oxygenation is mirrored in measurements of DBV and [dHb], which both result in a nonsignificant reduction in the amount of deoxyhaemoglobin present in the voxel.

## 4 DISCUSSION

Streamlined‐qBOLD is shown to provide metabolic information that is indicative of tissue viability following acute stroke. Detailed regional analysis demonstrates that *R*_2_ ′, DBV and [dHb] are sensitive to oxygenation related changes in tissues with varying outcomes, supported by serial‐imaging from example patient cases. Significant pairwise differences in voxel distributions were observed between the regional tissue ROIs using pairwise comparisons for *R*_2_ ′, DBV, and [dHb] (ischaemic core and contralateral tissue; infarct growth and contralateral tissue; ischaemic core and infarct growth; *p* < 0.01 in all cases). At the patient level, a significant difference between tissue outcomes was only observed for [dHb], with pairwise comparisons revealing this effect to be driven by a significant difference between ischaemic core and both infarct growth and contralateral tissue. Furthermore, serial analysis at the patient level revealed significant changes in *R*_2_ ′ between the presentation and 1 week scans for both ischaemic core (*p* < 0.01) and infarct growth (*p* < 0.05).

### 4.1 Ischaemic penumbra

The definition of the infarct growth region used in this study is expected to be spatially and metabolically consistent with the fraction of the ischaemic penumbra that does not survive. In this region, an increase in [dHb] is anticipated in order to maintain the rate of oxygen metabolism in tissue that is experiencing a reduction in CBF. The potential of sqBOLD to detect these changes was demonstrated by the statistically significant increase in [dHb] measured in the infarct growth ROIs at the voxel level (Figure 2 ; *p* < 0.01). Furthermore, [dHb] was found to be elevated, and CBF reduced, at the patient‐level, although this was not statistically significant (Figure 3). In addition to the observed changes in CBF and [dHb], DBV was found to be elevated in the infarct growth region (Figure 3). In order to interpret this it is important to understand the definition of DBV, which represents the blood volume occupied by deoxygenated blood. In healthy tissue this is largely contained within the veins and capillaries and an elevation would therefore normally result from passive inflation during increases in CBF. Since CBF is reduced here, increases in DBV can only be achieved by decreasing blood oxygenation in normally highly oxygenated vessels, such as precapillary arterioles. These vessels have already been shown to have a higher degree of desaturation than previously thought (Vovenko, 1999) which could feasibly be further reduced during ischaemia.

The spatial correspondence of the sqBOLD parameter maps with the infarct growth ROI can also be observed at the individual patient level (Figure 4 ). This example case shows presenting measures of *R*_2_′, DBV, and [dHb] that are elevated in regions that correspond to the infarct growth ROI. The CBF parameter map acquired on presentation demonstrates a large region of decreased flow that coincides with the elevated regions on the sqBOLD parameter maps. A restriction in flow is expected to result in an elevated OEF, which in turn causes an increase in the relative amount of deoxyhaemoglobin produced. As such, the observation of reduced CBF and elevated [dHb] in this patient is suggestive of the early identification of tissue exhibiting the physiological traits of the ischaemic penumbra. This opens up the prospect that concurrent MR based oxygenation and flow imaging can be used to identify tissue at risk of infarction (Astrup et al., 1981 ). In this patient, infarction occurs in this region at some point between the presenting and follow up scan times as evidenced by the infarct growth ROI (defined from the *b* = 1,000 s/mm ^2^ image). Therefore, early identification of penumbral tissue would provide a window of opportunity for interventions that might salvage this tissue.

### 4.2 Ischaemic core

From Figure 2, it can be seen that increases in all of the baseline brain oxygenation parameters are observed in the ischaemic core compared to infarct growth on presentation. This trend appears surprising at first, particularly if the elevated signal in the core is to be associated with the presence of deoxyhaemoglobin as a by‐product of ongoing metabolism. The infarct growth region is expected to contain tissue that is metabolically active on presentation but later recruited to the final infarct volume. This is in contrast to the nonviable tissue present in the ischaemic core. However, the elevated qBOLD signal measured in the ischaemic core can be explained either as stationary deoxyhaemoglobin in metabolically inactive regions with no blood supply or as ongoing metabolism in the diffusion lesion. This also offers two possible scenarios that explain the significant increase in core [dHb] measured at the patient‐level (Figure 3).

### 4.3 Stationary deoxyhaemoglobin

A similar regional trend in *R*_2_′ can be extrapolated from a previous study (Geisler et al., 2006 ) which looked at comparable tissue outcome ROIs. Here it was proposed that the elevated *R*_2_′ in the ischaemic core may result from stationary deoxyhaemoglobin present in vessels without blood supply. In the event of a complete occlusion of flow, stationary haemoglobin beyond the blockage will become fully deoxygenated as the remaining oxygen is metabolised leading to an increase in the amount of deoxyhaemoglobin present. This is likely to be the main contributing factor to the trend seen in Figure 2 , where the ischaemic core demonstrates the largest elevation in *R*_2_′, DBV, and [dHb]. In the context of the DBV elevation observed in the infarct growth region this would similarly reflect the desaturation of arteriolar blood vessels.

### 4.4 Reperfusion

In contrast, *R*_2_′ and [dHb] parameter maps in Figure 4 demonstrate a decrease in the ischaemic core. This may be explained by the presence or restoration of flow to an infarcted region. In this case, metabolically inactive tissue would not produce new deoxyhaemoglobin and previously produced deoxyhaemoglobin would be removed by the restored blood flow, leading to a decrease in *R*_2_′, DBV, and [dHb]. This patient received thrombolysis at 1 hr 24 min postonset. Despite this, flow is still noticeably reduced in this patient in the ischaemic core at presentation (Table 2) suggesting that recanalisation has not occurred. It is unclear whether this flow is sufficient to clear the deoxyhaemoglobin from metabolically inactive tissue.

### 4.5 Ongoing metabolism in the diffusion lesion

Elevated [dHb] and DBV in the ischaemic core may also be explained by observations made using PET, which have shown that regions of ongoing oxygen metabolism are possible within the presenting diffusion lesion (Fiehler et al., 2002; Guadagno et al., 2004, 2006; Kidwell et al., 2000 ). As such, it is possible that deoxyhaemoglobin production may still be occurring in regions of decreased ADC and contribute towards elevated *R*_2_ ′ in the ischaemic core. While none of the patients in this study clearly demonstrate this phenomenon (ie, elevated *R*_2_′ in the presence of nonzero CBF), the parameter maps shown in Figure 6 show some evidence of a heterogeneous pattern of blood oxygenation within the diffusion lesion. Unfortunately, these parameter maps are of low quality due to patient motion during the acquisition, particularly at the presenting time point. Head motion can introduce noise into the ASE *τ*‐series and can result in the failure of the FLAIR CSF nulling preparation. This has the potential to cause unreliable parameter fits. The impact of patient motion is considered later in this section.

### 4.6 Interpretability of [dHb] and OEF

It is evident that the relaxometry based method used in this study is sensitive to deoxyhaemoglobin regardless of the patency of the blood supply and can therefore exhibit elevated *R*_2_ ′ in the ischaemic core. As such, knowledge of the local blood supply is important to distinguish stationary deoxyhaemoglobin present in infarcted tissue from active tissue with an elevated metabolism. This motivated the calculation of [dHb] rather than OEF to avoid the false interpretation of a high *R*_2_′ as always representing elevated oxygen extraction. This sensitivity to stationary deoxyhaemoglobin also reconciles the apparent differences between PET and BOLD based measurements (Geisler et al., 2006 ). In PET the oxygen sensitive tracer is prevented from being delivered to the ischaemic core, meaning that signal is not detected there and reduced oxygen metabolism is inferred. This is in contrast to BOLD based measurements, which do not rely on the arrival of a tracer and hence the presence of deoxyhaemoglobin will still cause an increase in *R*_2_′. However, in this study all of the patients with hypoperfused lesions had nonzero median CBF in the ischaemic core ROI at presentation (Table 2 ). It remains to be seen whether there is a threshold CBF below which the blood is *functionally* stationary with respect to the accumulation of deoxyhaemoglobin.

### 4.7 Prospects for serial imaging of brain oxygenation

Streamlined‐qBOLD and ASL data were successfully acquired at each of the time points listed in Table 1 . Follow up time points were most commonly missed due to patient or scanner unavailablility. Despite this six out of the nine patients included in this study had a 1 week follow up scan. Comparison of sqBOLD and ASL metrics between presentation and 1 week at the patient level was performed by normalising these ROI based measurements to the contralateral ROI at each time point. This analysis revealed significant changes in *R*_2_ ′ for ischaemic core and infarct growth regions. This likely reflects the greater robustness of the *R*_2_′ maps compared to the physiological maps of DBV and [dHb] (Figure 1 ). Since *R*_2_ ′ reflects the amount of deoxyhaemoglobin present in the voxel, it can be seen that the decrease in DBV and [dHb] is consistent with a decrease in *R*_2_′. Likewise all three metrics converge towards unity at 1 week, representing a normalisation of these tissue oxygenation parameters. Conversely measurements of CBF become even greater than contralateral values at 1 week, representing increasing hyperperfusion.

### 4.8 Confounds: Non‐deoxyhaemoglobin related elevations and patient‐motion

Streamlined‐qBOLD is sensitive to other sources of magnetic susceptibility in the brain not related to deoxyhaemoglobin and care must be taken when interpreting elevations in signal. Iron and myelin are known sources of susceptibility that can confound the accurate quantification of brain oxygenation with this method and are of particular relevance as both can vary during ageing and in different pathologies. Figure 7 shows bilateral elevations in *R*_2_ ′ on both the affected and unaffected sides of the brain due to the high iron content of the deep grey matter structures. The presenting [dHb] map appears to be more highly elevated in deep grey matter on the affected side, pointing towards the importance of interpreting the *R*_2_′, DBV, [dHb], and CBF parameter maps in combination, as well as being aware of non‐oxygen related sources of susceptibility in the locality of the region of interest. Furthermore, a change in local haematocrit level could influence regional sqBOLD measurements (Broisat et al., 2018 ). For example, a local increase in Hct would increase *R*_2_′ even in the case of high CBF.

Significant head‐motion during imaging is a challenge in acute stroke patients. Despite the segmented nature of the FLAIR‐GASE acquisition, image artefacts were minimal. However, large head motions did impact the accuracy of the FLAIR CSF suppression. Slice selective inversion recovery pulses were used to optimise CSF suppression for each slice. However, large head motions between this FLAIR preparation and image acquisition resulted in ineffective removal of the CSF signal. This is evident in the presentation parameter maps in Figure 6 , where elevated signal can be seen within the ventricles, particularly in the *R*_2_ ′ map. As the presence of CSF signal can lead to apparent elevations in *R*_2_′ not related to oxygenation, it may obscure the oxygenation changes within the diffusion lesion on presentation (Dickson, Williams, Harding, Carpenter, & Ansorge, 2009; He & Yablonskiy, 2007; Simon, Dubowitz, Blockley, & Buxton, 2016). However, it is encouraging that heterogeneous patterns of oxygenation can be seen within the diffusion lesion at the follow‐up imaging time points where CSF suppression was effective. Furthermore, voxel‐wise error maps demonstrate that the error calculated on parameter estimates are considerably lower in tissue compared with values in the ventricles, where high DBV can be observed in some patients with high mean motion scores (Figure 1). This provides some assurance of oxygenation measurements made in tissue even in cases where there is significant head motion during sqBOLD acquisition. Through patient‐wise comparison of mean motion scores (Table 1 ) and *R*_2_′ standard deviation maps (Figure 1) it is noticeable that patients with higher motion scores demonstrate larger standard devaitions in areas such as the ventricles and subarachnoid spaces, consistent with a failure in CSF nulling. The impact of patient motion and elevated motion score on the parameter maps is demonstrated in Figure 4. Regions of reduced DBV and CBF are noticeable in the larger contralateral region on presentation. This is at least partially due to motion artefact as evidenced by the relatively high mean motion score (P03, Table 1). Although there are localised areas of decreased DBV and CBF in the presenting contralateral ROI, the median values from this region (P03, Table 1) are not unreasonably low.

From Figure 5 a noticeable increase in DBV can be seen between the presentation and 1 week follow‐up scans. On closer inspection, the 1 week DBV error map (data not shown) appears uniformly elevated compared to presentation. Further inspection of this data, which was found to have a low mean motion score, indicates that patient motion was not a problem here. It is therefore possible that the observed elevation in DBV could be of physiological origin. However, further work is required to establish the accuracy and repeatability of this technique. From Figure 1 it can be seen that the error in DBV can be quite high and from previous work it has been noted that the accuracy of this measurement requires improvement (Stone & Blockley, 2017).

### 4.9 Group heterogeneity, flow, and further work

From the patient‐level analysis (Figure 3 ), only the ischaemic core [dHb] demonstrated a significantly different value from infarct growth and contralateral ROIs. Although strict inclusion criteria were employed in this study to provide a uniform patient cohort, the failure to detect significant changes in *R*_2_′ and DBV may be partly explained by remaining heterogeneity in this group. Heterogeneity in regional perfusion status is apparent, with almost equal numbers of patients exhibiting hypo‐ and hyperperfusion in their presenting ischaemic core ROIs. A further source of heterogeneity across the group may be attributed to differences in onset to scan time (Table 1).

Alongside demonstrating the sensitivity of sqBOLD to changes in oxygenation, this study provides a demonstration of how complementary measurements of flow and oxygenation can be used to provide a unique insight into tissue viability. In practise, the information provided by sqBOLD could aid the interpretation of ASL CBF measurements, since low flow does not always progress to infarction in regions experiencing benign oligaemia (Kidwell, Alger, & Saver, 2003). Although it is beyond the scope of this study, the combination of CBF and [dHb] measurements allows for the calculation of the cerebral metabolic rate of oxygen consumption (CMRO_2_) (Blockley, Griffeth, Stone, Hare, & Bulte, 2015). This has been shown to improve tissue outcome prediction and may partly explain the variability seen in the presenting sqBOLD oxygenation measurements (An et al., 2015). The ASL acquisition used in this study utilises multiple postlabelling delays and as such offers improved accuracy of CBF quantification in the case of delayed blood arrival times. However, care should be taken when comparing these resuls with typical DSC PWI measurements where TTP and MTT are often preferred.

The identification of tissue outcomes based solely on measurements from this method does not currently appear to be possible, as evidenced by the considerable overlap between tissue outcome distributions in Figure 2. However, Kruskal–Wallis tests and post hoc pairwise comparisons found significant differences between these distributions suggesting that tissue outcome is dependent on tissue oxygenation and that the parameter maps derived from sqBOLD are sensitive to identifying this information on presentation. As such, sqBOLD provides complementary information to existing imaging modalities such as DWI and ASL and the combination of this information may allow for earlier identification of tissue under metabolic stress during the acute phases of stroke (An et al., 2015).

Future development of sqBOLD should focus on improving the robustness of the oxygenation measurements through refinement of the sqBOLD acquisition, modelling and analysis routines. This work would be aided by a study of the repeatability of the *R*_2_ ′ measurement. To date the only study to compare multiple techniques for measuring *R*_2_′ suggested that ASE has a marginally increased intersubject standard deviation (Ni, Christen, Zun, & Zaharchuk, 2015). However, it is difficult to compare these results with the current work, since the acquisitions differ significantly, and therefore further investigation of the GASE technique is warranted.

In addition, this study supports the further investigation of sqBOLD in a larger scale study and highlights the importance of controlling for onset to scan time and tissue perfusion status. The results presented here provide an initial estimate of the effect size that may be used to ensure follow on studies are adequately powered (Table 3). The noninvasive, quantitative nature of this method also means it is suitable for the longitudinal monitoring of stroke evolution and may provide unique insight into the various pathways to infarction and recovery, as well as providing valuable biomarkers with which to assess treatment and intervention (Figure 8).

## 5 CONCLUSION

Streamlined‐qBOLD was used to acquire information about oxygen metabolism in a cohort of acute ischaemic stroke patients, which is complementary to conventional MRI methodologies. It was found that resting brain oxygenation related parameters (*R*_2_ ′, DBV, and [dHb]) vary between regions with different tissue outcomes. The appropriate implementation of *R*_2_′, DBV and [dHb] parameter maps has the potential to refine the identification of the ischaemic penumbra.

## Supporting information

Appendix A.Supplementary data
The parameter maps and ROIs that underpin Figures 4 ‐ 7 can be accessed via the Oxford Research Archive repository, doi: https://doi.org/10.5287/bodleian:VYmwzrzpd alongside scripts which can be used to reproduce the charts in Figure 2 & 3 doi: https://doi.org/10.5281/zenodo.833474
