# Supplementary material for: Prospects for investigating brain oxygenation in acute stroke: Experience with a non‐contrast quantitative BOLD based approach
Source: Hum Brain Mapp. 2019 Mar 12;40(10):2853–66. doi: 10.1002/hbm.24564 (PMC6563088; doi:10.1002/hbm.24564)
Supplement: Supplementary file 1 — Appendix A.Supplementary data The parameter maps and ROIs that underpin Figures 4 ‐ 7 can be accessed via the Oxford Research Archive repository, doi: https://doi.org/10.5287/bodleian:VYmwzrzpd alongside scripts which can be used to reproduce the charts in Figure 2 & 3 doi: https://doi.org/10.5281/zenodo.833474 [file HBM-40-2853-s001.docx]

**Appendix A. Supplementary data**

The parameter maps and ROIs that underpin **Figures 4** - **7** can be accessed via the Oxford Research Archive repository, doi: <http://dx.doi.org/10.5287/bodleian:VYmwzrzpd> alongside scripts which can be used to reproduce the charts in **Figure 2 & 3** doi: <http://dx.doi.org/10.5281/zenodo.833474>
